# Salinity Tolerance, Ion Accumulation Potential and Osmotic Adjustment In Vitro and In Planta of Different *Armeria maritima* Accessions from a Dry Coastal Meadow

**DOI:** 10.3390/plants11192570

**Published:** 2022-09-29

**Authors:** Līva Purmale, Astra Jēkabsone, Una Andersone-Ozola, Gederts Ievinsh

**Affiliations:** Department of Plant Physiology, Faculty of Biology, University of Latvia, 1 Jelgavas Str., LV-1004 Rīga, Latvia

**Keywords:** *Armeria maritima*, electrical conductivity, functional differences, ion accumulation, non-ionic osmolytes, osmotic adjustment, potassium, salinity, sodium, tissue culture

## Abstract

The aim of the present study was to compare tolerance to salinity and ion accumulation potential of *Armeria maritima* subsp. *elongata*. Three accessions (AM1 and AM2, both from Latvia, and AM3 from Sweden) from relatively dry sandy soil habitats in the Baltic Sea region were selected and compared using both in vitro cultivated shoot explants and long-term soil-cultivated plants at flowering stage. Growth of root non-forming explants treated with increasing concentrations of NaCl was significantly inhibited starting from 110 mmol L^−1^, and the rate of shoot formation was even more sensitive. Significant differences in morphology and responses to salinity were found between different accessions. For soil-grown plants, biomass accumulation in above-ground parts was relatively little affected by salinity in AM1 and AM2 in comparison to that in AM3. Differences in ion accumulation were evident between the accessions as well as in respect to cultivation system used. Maximum accumulation capacity for Na^+^ was up to 2.5 mol kg^−1^ both in shoot explant tissues and in old leaves of soil-grown plants treated with NaCl, but that for K^+^ reached 4.0 mol kg^−1^ in old leaves of soil-grown plants treated with KCl. Non-ionic component of osmotic value was relatively high in old leaves and significantly increased under NaCl treatment, especially for AM2 and AM3 plants at moderate salinity, but in AM1 only at high salinity. In contrast, it significantly decreased in old leaves of AM2 plants treated with increasing concentration of KCl. It can be concluded that a wide salinity tolerance exists within *A. maritima* accessions from dry sandy soil habitats, associated with the ability to accumulate surplus ions both in salt glands and old leaves.

## 1. Introduction

Increasing soil salinity is one of the major threats to agricultural production, especially, in a light of global climate change-dependent increase in environmental heterogeneity [1,2,3]. Therefore, studies aiming to understand the mechanisms of salt tolerance are gaining special importance. In this respect, plant species native to salt-affected habitats are especially valuable models for understanding physiological characteristics of adaptive value important for life in saline environments [4].

In contrast to salt exclusion approach used by salt tolerant glycophytes, tight control of compartmentation of salinity-related ions is one of the adaptive mechanisms used by plants native to saline habitats [5]. As ionic species are both electrolytically and osmotically active, appropriate internal adjustment of osmotic balance is an important strategy for salinity tolerance. A major nonessential substance in soils of sea-water affected habitats is NaCl, but other types of soil salinity may be common in other situations [3]. Usually, when characterizing plant responses and resistance to salinity, emphasis is placed on Na^+^ toxicity in the form of NaCl. In the context of salinity, K^+^ has been analyzed mostly in a view of necessity to maintain high cytoplasmic K^+^/Na^+^ concentration ratio [6,7]. It was shown that coastal plant species from salt-affected habitats represent either Na^+^ excluders, regulating tissue electrical conductivity (EC) by changes in K^+^ concentration, K^+^ excluders, regulating EC by changes in Na^+^ concentration, or tight EC regulators [8]. There is a reason to suggest that for typical high salt-adapted species native to saline habitats (halophytes) high level of K^+^ in substrate will have the same negative effects than Na^+^ [9]. It was also shown that for halophytes, NaCl toxicity is related mainly to the effect of Cl^–^ [10].

Halophyte species, native to salt-affected habitats, represent a valuable resource for studies of physiological mechanisms related to salinity tolerance [11,12]. Usually the most common approach has been to use single halophytic model species or one halophyte and one glycophyte species [13], and to assess changes in different biochemical and physiological parameters, preferably over a gradient of salinity. Comparative studies of salinity tolerance have been performed recently involving a number of halophyte species from the same or similar habitats [14,15]. However, studies comparing different accessions or genotypes of a single halophyte species have been seldomly performed [16,17,18]. Recently, we performed a study involving a number of accessions of crop wild relative legume species *Trifolium fragiferum* and concluded that high intraspecies variability in morphological and physiological responses to salinity exist between geographically isolated populations [19].

Tissue culture has been often used as a tool for screening crop plant genotypes for their salinity tolerance [20,21,22]. In addition, it offers an opportunity to study salt tolerance-related responses at tissue level excluding whole plant-level responses, which can be important aspect of salinity tolerance [23]. However, in vitro studies of salinity tolerance with halophytic plant species have been relatively seldom performed. Thus, shoot explant culture was used for multiplication and selection of salt-tolerant genotypes of *Atriplex halimus* [24]. Axillary shoot culture was used to study antioxidative defense and osmotic adjustment of *Sesuvium portulacastrum* during high salinity [25]. Shoot cultures of *Salicornia europaea* [26], *Salicornia brachiata* [27] and *Limoniastrum monopetalum* [28] were used to reveal essentiality of NaCl for efficient in vitro propagation of these halophyte species. However, NaCl gradually reduced shoot proliferation in explants of *Crithmum maritimum* [29]. Several studies used seeds as explants to assess salinity tolerance of seedlings in tissue culture [30,31]. Comparative salinity tolerance studies using both tissue culture and whole plants grown in the substrate are rarely carried out [32].

Rosette-forming evergreen perennial species *Armeria maritima* (Mill.) Willd. (Plumbaginaceae) is characteristically found in temperate open habitats with dry, saline, sandy or metal-rich soil [33]. In a study with *A. maritima* plants from eight populations representing five different habitats in Britain (mountain, sea-cliff, salt marsh, shingle, and pasture) it was found that each population showed different ecological responses viewed as a result of local adaptation [34]. However, some traits of *A. maritima* exhibit phenotypic plasticity [33]. Presence of *A. maritima* in salt-affected habitats raises the question of salt tolerance of the species and its mechanisms. Previously, all ecotypes of *A. maritima* from different habitats have shown relatively high salinity tolerance, similar to that of species from brackish conditions of upper salt marsh, and even shoot growth stimulation at low salinity [35]. However, it is still an open question whether salinity tolerance of *A. maritima* plants from different populations is related to phenotypic plasticity or local genetic adaptation. In this respect, it is reasonable to ask whether there are differences between *A. maritima* accessions growing in habitats with relatively similar conditions but being geographically isolated, in respect to their responses to salinity. Recently we performed a comparative study of heavy metal tolerance and metal accumulation potential of three geographically isolated *A. maritima* accessions from a dry coastal meadow, and showed significant species-wide metal tolerance and extremely high accumulation potential of *A. maritima* but with some accession-specific differences [36]. Therefore, the aim of the present study was to analyze salinity tolerance of the three *A. maritima* subsp. *elongata* accessions from a dry coastal meadow in controlled conditions. A special attention was paid to possible different growth responses and ion accumulation potential to variable treatment with NaCl or KCl. It was hypothesized that similar effect of Na^+^ and K^+^ on growth of *A. maritima* plants will be found.

## 2. Materials and Methods

### 2.1. Plant Material

Seeds of *A. maritima* subsp. *elongata* from three geographically isolated micropopulations growing on sandy soils in water reservoir-associated meadows were used as propagation material (Table 1). Two small micropopulations in Latvia were located on a shore meadow of River Vecdaugava (AM1) and a shore meadow of Buļļupe (AM2). Micropopulation from Sweden (AM3) was located on a coastal meadow of the Baltic Sea. Seeds of AM1 and AM2 were used for initiation of tissue culture as described further. Multiplied shoot explants were used for tissue culture experiment or were rooted and acclimatized for soil culture experiment. Seeds of AM3 were used for establishment of plants for soil culture experiment. Salt treatments for soil-grown plants were performed at the beginning of appearance of reproductive structures.

Soil electrical conductivity (EC) was measured in natural habitats using HH2 m equipped with WET-2 sensor (Delta-T Devices, Burwell, UK) at five spots at least 5 m apart. For all micropopulations, soil was non-saline or slightly saline (Table 1).

### 2.2. In Vitro Experiment

Seeds of AM1 and AM2 were used as explants for establishment of tissue culture. All details on culture initiation and multiplication were as described previously [36]. Briefly, Murashige and Skoog medium supplemented with 30 g L^−1^ sucrose and 6 g L^−1^ agar was used for initial culture, followed by multiplication on Murashige and Skoog medium containing sucrose and agar supplemented with 0.1 mg L^−1^ 1-naphthaleneacetic acid and 1 mg L^−1^ 6-benzylaminopurine. Rooting was performed on the same medium with 0.2 mg L^−1^ 1-naphthaleneacetic acid. Rooted explants were acclimatized ex vitro in a peat substrate for two weeks and further used for in planta experiment.

Shoot explants at multiplication stage were used for treatment with NaCl. Necessary concentration of NaCl (Table 1) was added to multiplication medium before autoclaving. Medium was poured in 200 mL jars (five per treatment) and five shoot explants were placed in each jar. Cultures were placed in a growth cabinet under 16 h photoperiod provided by a fluorescent light with photon flux density 50 μmol m^−2^ s^−1^ of photosynthetically active radiation at 25 °C. After 4 weeks, the experiment was terminated. Multiplication rate was evaluated by counting number of shoots per explant. Fresh and dry mass (after drying at 60 °C for 72 h) of tissues were measured. Tissue water content was expressed as g of H_2_O per g dry mass.

### 2.3. In Planta Experiments

For accessions AM1 and AM2, rooted and acclimatized explants were used for establishment of experimental material as described previously [36]. Plants were individually planted in 1.3 L plastic containers filled with 1 L of a mixture of quartz sand (Saulkalne S, Saulkalne, Latvia) and heat-treated (60 °C, 24 h) garden soil (Biolan, Eura, Finland) 1:3 (*v*/*v*), and placed in an experimental automated greenhouse (HortiMaX, Maasdijk, Netherlands) with supplemented light from Master SON-TPIA Green Power CG T 400 W (Philips, Amsterdam, Netherlands) and Powerstar HQI-BT 400 W/D PRO (Osram, Munich, Germany) lamps (photon flux density of photosynthetically active radiation 380 µmol m^−2^ s^−1^ at the plant level), 16 h photoperiod, day/night temperature 24/16 °C, relative air humidity 60 to 70%. Salinity treatment (Table 1) was started after a week-long period of additional acclimatization in greenhouse. Salt treatment was performed gradually, by 44 mmol L^−1^ increments during 5 weeks, using NaCl and KCl solution. Necessary amount of salt was dissolved in deionized water and 0.1 L per container was applied to soil. During treatments, plants started to develop generative structures. Plants were cultivated for 8 more weeks after reaching full treatment.

Plant material for accession AM3 was established by seeds as described previously [36]. Seeds were surface sterilized with a half-diluted commercial bleach (ACE, Procter & Gamble, Warszawa, Poland) and sown in 1 L plastic plant tissue culture containers filled with autoclaved (1 atm, 20 min) garden soil (Biolan, Eura, Finland), closed with lids and further cultivated for two weeks in a growth cabinet (light/dark period of 16/8 h, photosynthetically active radiation with a photon flux density 100 µmol m^−2^ s^−1^, day/night temperature 5/15 °C). After change of temperature regime to day/night temperature 15/20 °C for two additional weeks, seedlings were transplanted to 0.25 L plastic containers with a mixture of quartz sand (Saulkalne S, Saulkalne, Latvia) and garden soil (Biolan, Eura, Finland) 1:3 (*v*/*v*) and acclimatized to greenhouse conditions. Final transplantation to 0.5 L plastic containers with the same substrate was performed after two weeks. Fully developed 2-month-old plants in a vegetative stage were assigned to one of 12 treatments (Table 1), five individual plants per treatment. Salt treatment was performed gradually, by not more than 44 mmol L^−1^ increments during 5 weeks, using NaCl and KCl solution. During treatments, plants started to develop generative structures. Plants were cultivated for 7 more weeks after reaching full treatment.

During cultivation, individual containers were randomly redistributed weekly on a greenhouse bench. Substrate water content was monitored with HH2 moisture meter equipped with WET-2 sensor (Delta-T Devices, Burwell, UK) and kept at 50 to 60%. Every third week plants were fertilized with Yara Tera Kristalon Red and Yara Tera Calcinit fertilizers (Yara International, Oslo, Norway). A stock solution was prepared for each fertilizer (100 g L^−1^) and working solution contained 25 mL of each per 10 L deionized water, used with a rate 100 mL per container.

At termination of each experiment, plants were individually separated in different parts (roots, flower stalks, inflorescences (flowers), leaves of different age). In experiment with AM1 and AM2, older leaves did not fully decay, as only part of the particular leaf was becoming brown and dry. Therefore, instead of designating these leaves as “decayed”, all leaves were sorted either as “old” or “young” according to their position and general morphological appearance. In experiment with AM3, individual older leaves decayed fully, therefore, all leaves were sorted either as “decayed” or “living” but for sake of comparison were indicated as “old” or “young”, respectively. Inflorescences were counted, and the length of flower stalks was measured. Plant material was weighed separately before and after drying in an oven at 60 °C for 72 h. Water content was calculated as g H_2_O per g dry mass.

### 2.4. Measurements

All analyses were performed in triplicate, using representative tissue samples from individual biological replicates. Plant tissues were homogenized by crushing and a sample (0.2 g) was taken for analysis of EC, Na^+^ concentration and K^+^ concentration in water extract by respective compact meters and analysis of osmotic activity by a freezing point osmometer. Tissues were ground with mortar and pestle to a fine powder and 10 mL of deionized water was added. The homogenate was stirred with pestle for 1 min. After filtration through nylon mesh cloth (No. 80) homogenate was used for measurement of ion concentration by LAQUAtwin compact meters B-722 (Na^+^) and B-731 (K^+^), and electrical conductivity by LAQUAtwin conductivity meter B-771 (Horiba, Kyoto, Japan) and measurement of osmotic value. For osmotic value analysis, 50 μL of extract were transferred in a 1.5 mL Eppendorf tube and placed in a freezing point osmometer Osmomat 3000 Basic (Gonotec Meβ- und Regeltechnik, Berlin, Germany) and operated according to the manufacturer’s instructions. Using a standard curve for different concentrations of NaCl and KCl, the osmotic value caused by the total concentration of Na^+^ and K^+^ was calculated according to the actual Na^+^ and K^+^ concentration of each sample extract. For each sample, the difference between the total osmotic value and the osmotic value due to Na^+^, K^+^ and Cl^–^ ions was calculated and designated as “non-ionic osmotic value”, which showed the osmotic effect of other osmotically active ions (besides Na^+^, K^+^ and Cl^–^) or non-ionic compounds. At least three analytical replicates were performed for each sample and the average value was calculated.

### 2.5. Data Analysis

Results were analyzed by KaleidaGraph (v. 5.0, Synergy Software, Reading, PA, USA). Statistical significance of differences was evaluated by one-way ANOVA using post-hoc analysis with minimum significant difference. Significant differences were indicated at *p* < 0.05.

## 3. Results

### 3.1. In Vitro Experiment

**Figure 1:**
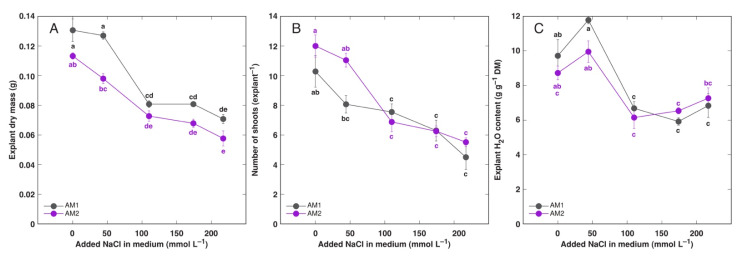
Effect of added Na concentration in tissue culture medium on dry mass (**A**), number of shoots (**B**) and tissue water content (**C**) of explants of *Armeria maritima* accessions AM1 and AM2 after 4 weeks of cultivation. Data are means ± SE from 5 replicates, with five explants each. Different letters of respective color between accessions and treatments indicate statistically significant differences (*p* < 0.05).

Growth and proliferation of root non-forming explants of *A. maritima* cultivated on multiplication medium was negatively affected by increasing concentration of NaCl in the medium (Figure 1). Biomass of explants from accession AM1 tended to be higher at all NaCl concentrations in comparison to AM2, but significant difference was evident only at 44 mmol L^−1^ (Figure 1A). At the highest NaCl concentration (217 mol L^−1^) biomass accumulation was inhibited by 46 and 49%, and proliferation by 56 and 54%, for AM1 and AM2, respectively. Water content in explants showed a tendency to increase at 44 mmol L^−1^ NaCl, followed by a significant decrease at 110 mmol L^−1^ (Figure 1C). Further increase in NaCl concentration did not result in changes of water content.

**Figure 2:**
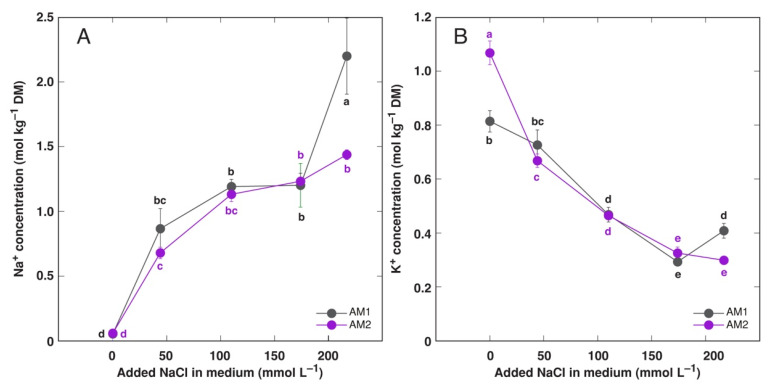

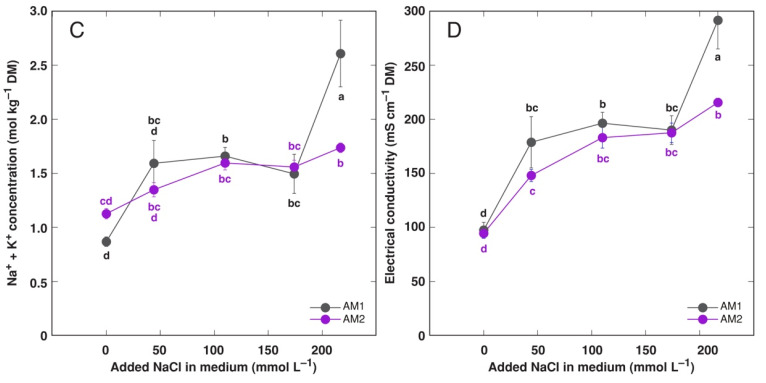
Effect of added Na^+^ concentration in tissue culture medium on Na^+^ concentration (**A**), K^+^ concentration (**B**), summed Na^+^ + K^+^ concentration (**C**) and electrical conductivity (**D**) in explant tissues of *Armeria maritima* accessions AM1 and AM2 after 4 weeks of cultivation. DM, dry mass. Data are means ± SE from 4–5 replicates. Different letters of respective color between accessions and treatments indicate statistically significant differences (*p* < 0.05).

Concentration of Na^+^ increased in explant tissues cultivated in presence of increasing medium NaCl, but the accumulation response seemed to be saturable at 110 mmol L^−1^ (Figure 2A). However, at the highest medium NaCl concentration, explants of AM1 showed significant further increase in tissue Na^+^ concentration. In contrast, explant K^+^ concentration decreased with increasing medium NaCl concentration up to 174 mmol L^−1^ (Figure 2B). As a result, summed Na^+^ + K^+^ concentration was relatively stable over a range of medium NaCl concentration (Figure 2C). However, increase in tissue electrical conductivity with increasing salinity was relatively more pronounced (Figure 2D).

### 3.2. In Planta Experiments: Effect of Salinity on Growth

**Figure 3:**
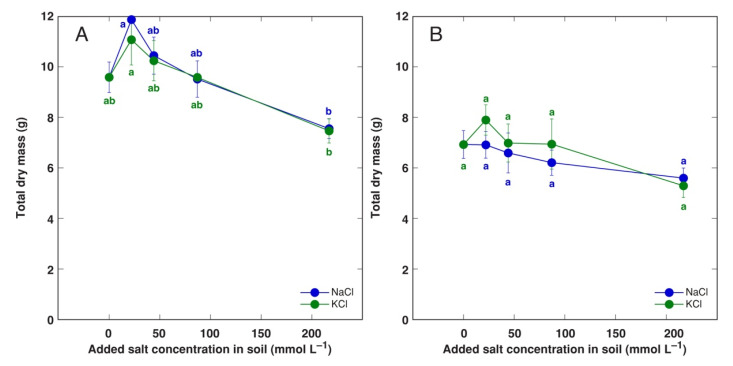
Effect of added NaCl and KCl concentration in soil on total dry mass of *Armeria maritima* plants from accessions AM1 (**A**) and AM2 (**B**) after 8 weeks of cultivation. Data are means ± SE from 5 replicates. Different letters of respective color between accessions and treatments indicate statistically significant differences (*p* < 0.05).

Effect of Na^+^ and K^+^ in a form of chloride on growth of *A. maritima* plants cultivated in substrate was identical. Total biomass of substrate-cultivated plants for accessions AM1 and AM2 was relatively stable over increasing NaCl and KCl concentration range, and no significant decrease was evident even at 217 mol L^−1^ salinity (Figure 3). However, as biomass tended to increase for AM1 plants cultivated at 22 mol L^−1^, there was a significant difference between plants at 22 and 217 mL L^−1^ (Figure 3A).

Number of flower stalks and dry mass of leaves (Table 2), as well as dry mass of both flower stalks and flowers was not significantly affected by increasing salinity for both AM1 and AM2 (Table 3). However, total length of flowers talks was significantly decreased for AM2 at 217 mol L^−1^ KCl (Table 2). Biomass of roots was significantly decreased for AM1 at 87 and 217 mmol L^−1^ salinity for both NaCl- and KCl-treated plants, but was not significantly affected for AM2 plants (Table 3).

**Figure 4:**
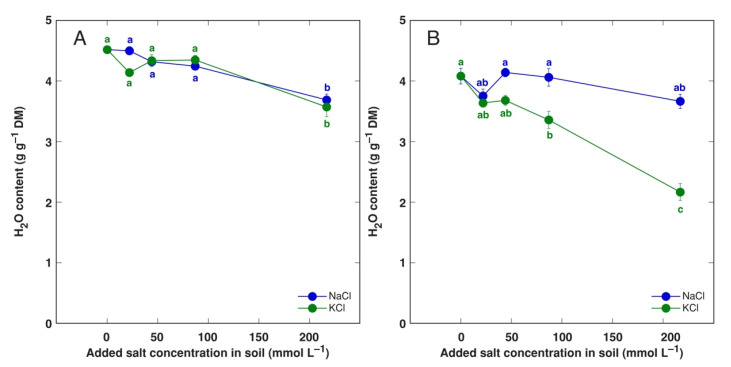
Effect of added NaCl and KCl concentration in soil on water content in old leaves of *Armeria maritima* plants from accessions AM1 (**A**) and AM2 (**B**) after 8 weeks of cultivation. DM, dry mass. Data are means ± SE from 5 replicates. Different letters of respective color between accessions and treatments indicate statistically significant differences (*p* < 0.05).

Significant decrease in water content of old leaves of accession AM1 at 217 mmol L^−1^ salinity for both NaCl- and KCl-treated plants indicated similar degree of stimulation of leaf decay (Figure 4A). However, for plants from accession AM2, the degree of decay was significantly more pronounced for plants treated with 87 and 217 mol L^−1^, in comparison to NaCl-treated plants (Figure 4B).

**Figure 5:**
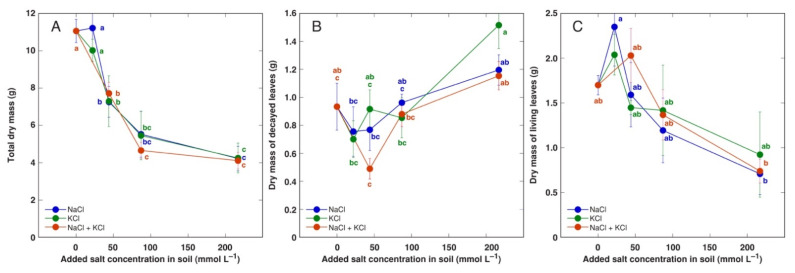
Effect of added NaCl, KCl and NaCl + KCl concentration in soil on total dry mass (**A**), dry mass of decayed leaves (**B**) and dry mass of living leaves (**C**) of *Armeria maritima* plants from accession AM3 after 7 weeks of cultivation. Data are means ± SE from 5 replicates. Different letters of respective color between accessions and treatments indicate statistically significant differences (*p* < 0.05).

*A. maritima* plants of accession AM3 was treated with identical concentrations of NaCl, KCl or combination of NaCl with KCl, and all treatments had identical negative effect on total biomass starting from 44 mol L^−1^ (Figure 5A). Biomass of decayed leaves had a tendency to increase at 217 mol L^−1^ salinity, especially, for KCl-treated plants, but the effect was not statistically significant (Figure 5B). Biomass of living leaves tended to increase at low salinity and decreased with increasing salt concentration, but the effect was not statistically significant due to high variability between individual plants (Figure 5C). As a result, total dry mass of leaves did not change significantly in salt-treated AM3 plants (Table 4). However, growth of reproductive structures and roots was more negatively affected by increasing salinity (Table 4). Thus, number of flower stalks significantly decreased from 87 mml L^−1^ salinity, total length and dry mass of flower stalks significantly decreased from 44 mol L^−1^ salinity, but dry mass of flowers significantly decreased from 217 mmol L^−1^ for NaCl, 44 mmol L^−1^ for KCl and NaCl + KCl treatments. Root biomass tended to decrease from 44 mmol L^−1^, and the effect was not statistically significant for NaCl, but was significant starting from 87 mmol L^−1^ for plants treated with KCl and NaCl + KCl.

### 3.3. In Planta Experiments: Effect of Salinity on Ion Accumulation and Osmotic Adjustment

**Figure 6:**
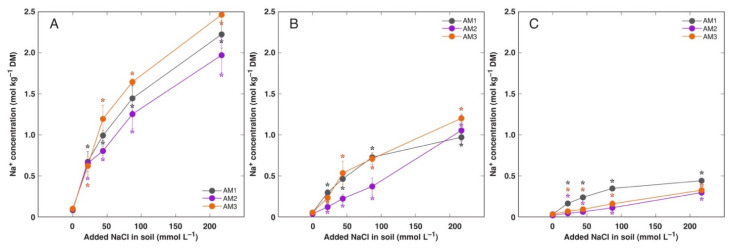
Effect of added NaCl concentration in soil on accumulation of Na^+^ in old leaves (**A**), new leaves (**B**) and flowers (**C**) of *Armeria maritima* plants from different accessions after 7–8 weeks of cultivation. DM, dry mass. Data are means ± SE from 3 replicates. Asterisks of respective color indicate statistically significant differences (*p* < 0.05) from control.

**Figure 7:**
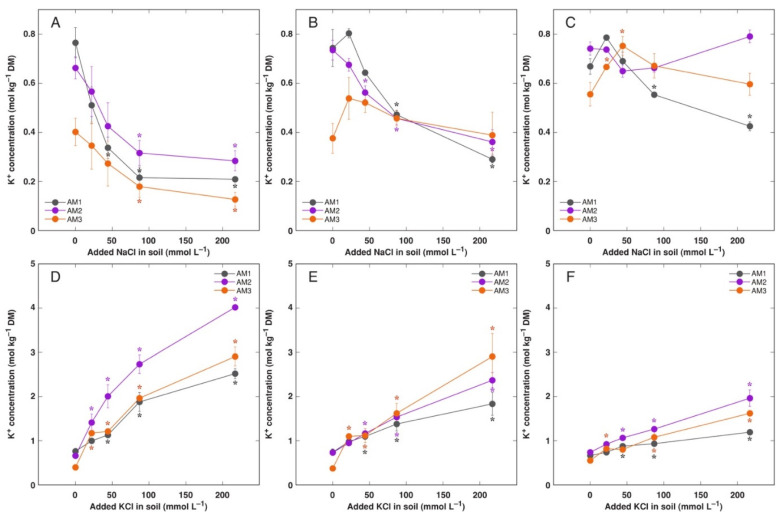
Effect of added NaCl (**A**–**C**) and KCl (**D**–**F**) concentration in soil on accumulation of K^+^ in old leaves (**A**,**D**), new leaves (**B**,**E**) and flowers (**C**,**F**) of *Armeria maritima* plants from different accessions after 7–8 weeks of cultivation. DM, dry mass. Data are means ± SE from 3 replicates. Asterisks of respective color indicate statistically significant differences (*p* < 0.05) from control.

Possible differences in accumulation of electrolytically and osmotically active ions Na^+^ and K^+^ in *A. maritima* plants from three different accessions treated with NaCl or KCl were evaluated after long-term cultivation in substrate. Plants treated with 217 mmol L^−1^ NaCl as well as plants treated with 87 and 217 mmol L^−1^ KCl actively deposited salt crystals on surface of leaves and flower stalks (Figure S1), but intensity of deposition was not estimated. Nevertheless, growth at increased substrate salinity caused either by NaCl or KCl resulted in increased accumulation of respective cations in all plant parts, but with clear organ specific pattern of accumulation (Figure 6, Figure 7D–F and Figures S2 and S3). The highest Na^+^ concentration reached was 2.48 mol kg^−1^ (57 g kg^−1^) in old leaves of plants from accession AM3 treated with 217 mmol L^−1^ NaCl (Figure 6A), and that for K^+^ was 4.03 mol kg^−1^ (157 g kg^−1^) in old leaves of plants from accession AM2 treated with 217 mmol L^−1^ KCl (Figure 7D). Accumulation capacity of either Na^+^ or K^+^ in plants treated with NaCl and KCl, respectively, increased in an order roots < flowers < flower stalks < new leaves < old leaves. There was a stable tendency that AM3 plants accumulated more Na^+^ in old leaves of NaCl-treated plants in comparison to AM1 and AM2 plants (Figure 6A), and AM2 plants accumulated more K^+^ in old leaves of KCl-treated plants in comparison to AM1 and AM3 plants (Figure 7D). It is also important that accumulation capacity for K^+^ in all plant parts was higher than that for Na^+^ at identical salinity caused either by KCl or NaCl treatment, respectively.

For NaCl-treated plants, increased salinity resulted in a significant reduction in K^+^ concentration in old leaves for all accessions (Figure 7A) and new leaves for AM1 and AM2 (Figure 7B). Effect of NaCl treatment on K^+^ concentration in generative parts and roots differed between accessions. Thus, it significantly increased in flowers (Figure 7C) and flower stalks (Figure S3B) of plants from accession AM3, but decreased in flowers of AM1 (Figure 7C) and flower stalks of AM1 and AM2 (Figure S3B). Moreover, K^+^ concentration tended to decrease in roots of NaCl-treated AM1 and AM2 plants (Figure S3A).

**Figure 8:**
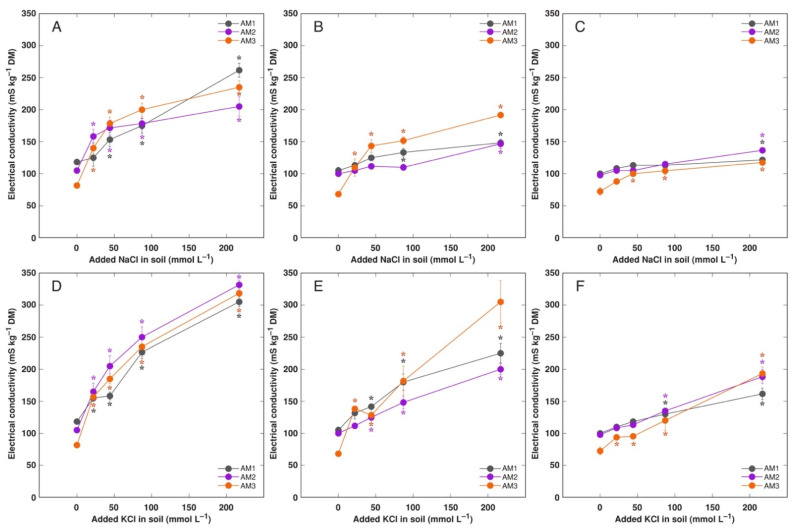
Effect of added NaCl (**A**–**C**) and KCl (**D**–**F**) concentration in soil on electrical conductivity in old leaves (**A**,**D**), new leaves (**B**,**E**) and flowers (**C**,**F**) of *Armeria maritima* plants from different accessions after 7–8 weeks of cultivation. DM, dry mass. Data are means ± SE from 3 replicates. Asterisks of respective color indicate statistically significant differences (*p* < 0.05) from control.

Level of intensity of electrical conductivity was partitioned in *A. maritima* in a same way as Na^+^ and K^+^, and it tended to be higher in KCl-treated plants in comparison to the NaCl-treated plants (Figure 8 and Figure S4). Distribution and levels of summed Na^+^ + K^+^ concentration showed the similar character (Figure S5). Control plants of *A. maritima* showed pronounced gradient in K^+^: Na^+^ concentration ratio between plant parts, decreasing in an order flowers > new leaves > flower stalks > old leaves > roots, and plants from accession AM2 had higher values of the ratio in generative parts and new leaves (Figure S6).

**Figure 9:**
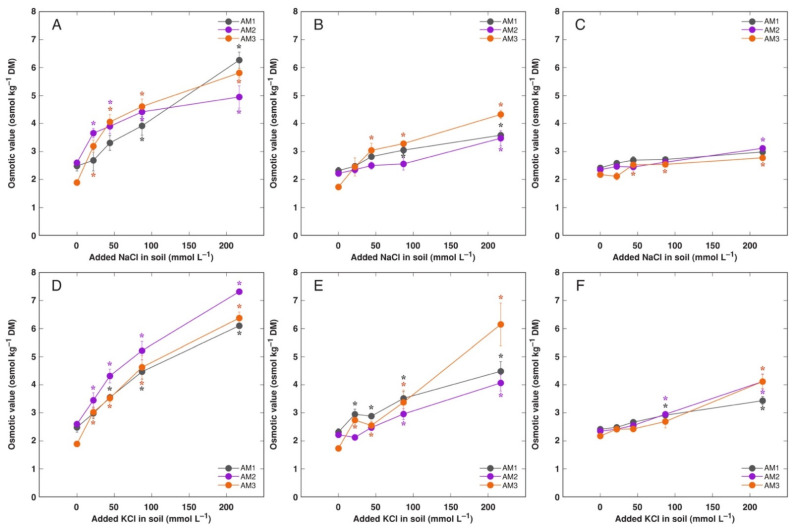
Effect of added NaCl (**A**–**C**) and KCl (**D**–**F**) concentration in soil on osmotic value in old leaves (**A**,**D**), new leaves (**B**,**E**) and flowers (**C**,**F**) of *Armeria maritima* plants from different accessions after 7–8 weeks of cultivation. DM, dry mass. Data are means ± SE from 3 replicates. Asterisks of respective color indicate statistically significant differences (*p* < 0.05) from control.

**Figure 10:**
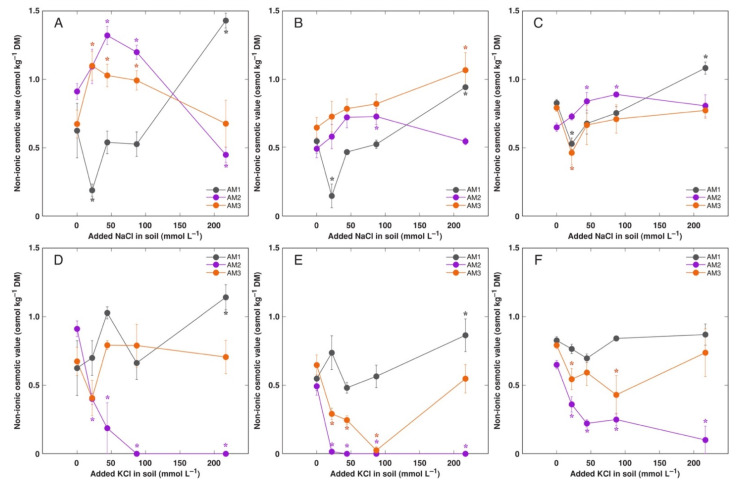
Effect of added NaCl (**A**–**C**) and KCl (**D**–**F**) concentration in soil on non-ionic osmotic value in old leaves (**A**,**D**), new leaves (**B**,**E**) and flowers (**C**,**F**) of *Armeria maritima* plants from different accessions after 7–8 weeks of cultivation. DM, dry mass. Data are means ± SE from 3 replicates. Asterisks of respective color indicate statistically significant differences (*p* < 0.05) from control.

Total osmotic value in *A. maritima* tissues under rising salinity increased in all plant parts in a concentration-dependent manner, but the increase was relatively less pronounced than that for electrical conductivity (Figure 9 and Figure S7). The highest values were reached in old leaves followed by new leaves and generative parts, and was the lowest in roots. There were no pronounced differences in osmotic value between various accessions, but it tended to be higher in some combinations of KCl-treated plants in comparison to NaCl-treated plants. However, there were pronounced differences in non-ionic osmotic values between plant parts, accession and treatments (Figure 10 and Figure S8). In old leaves of AM2 and AM3 plants treated with NaCl, non-ionic osmotic value significantly increased at low to moderate salinity, but decreased at high salinity (Figure 10A). However, for AM1, there was initial decrease of non-ionic osmotic value at low salinity, followed by increase at high salinity. In contrast, for KCl-treated plants of AM2, non-ionic osmotic value decreased with increasing salinity and completely disappeared at moderate and high salinity, while it did not significantly change for AM3 and increased for AM1 at high salinity (Figure 10D). For new leaves, non-ionic osmotic value tended to increase in AM2 plants at moderate salinity, and significantly increased in AM1 and AM3 plants at high salinity, when NaCl treatment was taken into account (Figure 10B). In contrast, in new leaves of KCl-treated plants, non-ionic osmotic value decreased in AM2 and in part in AM3, followed by recovery in high salinity, and increased in high salinity for AM1 (Figure 10E). Contrasting pattern in respect to non-ionic osmotic value was evident also for flowers between NaCl-treated (Figure 10C) and KCl-treated (Figure 10F) plants, as well as flower stalks (Figure S8B,D). In roots, non-ionic osmotic value increased at high salinity of AM1 plants at both treatments, but there was no non-ionic osmotic activity present in roots of AM3 (Figure S8A,C).

**Figure 11:**
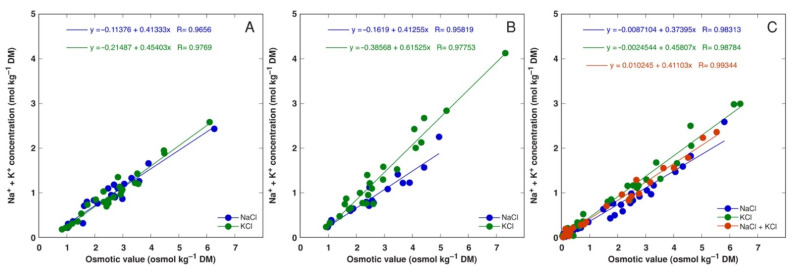
Relationship between Na^+^ + K^+^ concentration and osmotic value in all parts of *Armeria maritima* accessions AM1 (**A**), AM2 (**B**) and AM3 (**C**) treated with different salts. DM, dry mass.

Relationships between summed Na^+^ + K^+^ concentration and osmotic value in tissues confirmed differences in osmotic regulation between NaCl and KCl treatments in plants from accession AM2 and, partially, for AM3 (Figure 11).

## 4. Discussion

### 4.1. Salinity Tolerance of A. maritima

Given high ecological variability of *A. maritima* accessions, two earlier studies addressed question on possible differences in adaptations of *A. maritima* to high soil salinity between different geographically and ecologically isolated micropopulations [35,37]. In general, both salt marsh and inland populations of *A. maritima* showed relatively high salinity tolerance, possibly related to the ability of plants to allocate Na^+^ to leaves and to accumulate organic osmolytes. However, root growth of inland plants was extremely sensitive to salinity and leaf biomass significantly decreased by increasing salinity in contrast to salt marsh plants. Another difference was that only plants from a salt marsh habitat accumulated more Na^+^ in leaves in comparison to that in roots. Still, the question on whole-species abiotic stress tolerance vs. local genetic adaptation of *A. maritima* has not been definitively solved. Recently we have shown that the same accessions of *A. maritima* used also in the present study native to uncontaminated soil are highly tolerant to heavy metals and have an extreme metal accumulation ability, exceeding threshold concentration values for hyperaccumulation of Cd, Cu, Mn, Pb, and Zn [36]. As a next step, within the present study, we concentrated on characterization of ion distribution and osmotic protection of *A. maritima* plants under the effect of Na^+^ and K^+^ salinity.

According to the typology used for coastal plant species in respect to control of total concentration of electrolytically-active ions [8], *A. maritima* can be designated as EC controlling species, with both Na^+^ and K^+^ internal concentration concomitantly changing to keep relatively stable tissue concentration of soluble ions. Based on characteristic accumulation of ions in leaves and exclusion of them from roots and partially from reproductive structures, *A. maritima* can be characterized as salt-accumulating halophyte. Increased succulence is usually regarded as one of the mechanisms of salinity tolerance in Na^+^-accumulating halophyte species allowing to dilute salts and efficiently decrease salt concentration in tissues on a tissue water basis [38]. Thus, both NaCl and KCl treatments increased water content in leaves of halophyte *Atriplex halimus* at moderate salinity [39]. However, this was not the case with *A. maritima*. This difference is most probably related to the fact that *A. maritima* is a salt-secreting species, but it only secretes about 4% of the absorbed Na^+^ [40], thus, it can be characterized as salt-accumulating recretohalophyte.

Perennial plants with rosette type of growth are able for indeterminate production of new leaves from multiple apical meristems, allowing for induction of senescence and replacement of older leaves [41]. This mechanism seems to be especially important for evergreen species *A. maritima*, allowing deposition of harmful compounds in older leaves together with physiological exclusion of them from actively photosynthesizing leaves and reproductive structures. This phenomenon has been reflected in preferential accumulation of both heavy metals [36,42] and Na^+^ [35] in decaying leaves of *A. maritima*, and confirmed also by the results of the present study.

### 4.2. Differences between Accessions

In the earlier study, an ability to tolerate salinity was compared for sandy soil, heavy metal and salt marsh ecotypes of *A. maritima* cultivated in artificial soil in controlled conditions [35]. Low salinity (40 and 100 mM NaCl in soil solution) stimulated biomass accumulation in leaves for all ecotypes. However, root growth of sandy soil and heavy metal ecotypes was inhibited even at low salinity. High salinity (150 and 200 mM NaCl) resulted in growth inhibition of plants from both sandy soil and heavy metal populations, but growth of plants (both shoots and roots) from salt marsh population was not affected. In the present study, growth of plants from accessions AM1 and AM2 was not significantly affected by increasing salinity (Figure 3), but that of plants from accession AM3 was significantly inhibited already at 50 mmol L^−1^ (Figure 5A). Another difference between salt-adapted and inland populations was that only salt marsh plants accumulated more Na^+^ in green leaves in comparison to roots, and only at moderate to high salinity, but Na^+^ accumulation ability in leaves was similar for plants from all ecotypes, reaching 0.6–0.9 mol kg^−1^ at 200 mM NaCl [35]. In the present study, all accessions of *A. maritima* preferentially accumulated Na^+^ in above-ground parts, especially, leaves (Figure 6 and Figure S2), and Na^+^ concentration in young leaves reached 1.0–1.3 mol kg^−1^, with no significant differences between the accessions. In comparison, the potential for Na^+^ accumulation in proliferating explants in tissue culture reached 2.0 mol kg^−1^ (Figure 2A) showing very high tissue tolerance to Na^+^. Interestingly, Na^+^ concentration in decayed leaves and inflorescences was not ecotype-dependent and was 0.7–1.0 and 0.1–0.4 mol kg^−1^, respectively [35]. Consequently, according to the morphological responses and ion accumulation characteristics in conditions of increasing salinity, *A. maritima* accessions from sandy soil salt-unaffected habitats used in the present study have large similarities with these of salt-marsh specific ecotype of *A. maritima* studied earlier [35].

Leaves of plants from salt-marsh population had higher K^+^ concentration in control conditions, and it decreased with increasing salinity [35]. Similar decrease was evident for plants from sandy-soil population, but K^+^ concentration in leaves was stimulated by low to moderate salinity for plants from heavy-metal population. Similarly, K^+^ concentration significantly decreased in explant tissues (Figure 2B) as well as in leaves (Figure 7A,B) and flower stalks (Figure S3B) of soil-grown plants with increasing salinity.

### 4.3. Effect of Na^+^ vs. K^+^

Traditionally, Na^+^ is opposed to K^+^ in the manner of the classical battle of “evil” and “good” [6]. Of course, it is not debatable that K^+^ is an essential element for all plant species, but Na^+^ is necessary only for certain C_4_ species. However, “toxicity” of Na^+^, while widely advertised, has not been much experimentally proven, especially, in comparison to that of K^+^, and in salt-adapted species. As it has been correctly pointed out by Kronzucker et al. (2013) [43], “... several leading paradigms in the field, such as on the roles of Na^+^ influx and tissue accumulation or the cytosolic K^+^/Na^+^ ratio in the development of toxicity, are currently insufficiently substantiated and require a new, critical approach”.

One of the most important finding of the present study was that effect of Na^+^ and K^+^ in a form of chloride salt on growth of *A. maritima* plants was nearly identical. However, there were differences in water content in older leaves as well as in osmotic adjustment between the two cations. First, leaves of accession AM2 showed decreasing water content with increasing KCl concentration, reflecting increase of partial dieback of these leaves (Figure 4B). Second, treatment with KCl did not induce increase of non-ionic osmotic values in old leaves (Figure 10D) in comparison to that with NaCl (Figure 10A).

Evidence for equal effects of Na^+^ and K^+^ on plant growth contradicts the generally accepted view of Na^+^ as a particularly toxic chemical element in contrast to K^+^. While both elements have similar chemical properties and remain in a soluble form in plant cells, K^+^ is an essential plant nutrient with important physiological functions. For typical glycophyte species, effect of Na^+^ is characteristically more negative in comparison to that of surplus K^+^ [44]. However, for several halophyte species, KCl has the same of even more pronounced negative effect on plant growth in comparison to that of NaCl, as for *Atriplex nummularia* [9], *Sesuvium portulacastrum* [45], *Atriplex halimus* [39].

It appears that there are no Na^+^-specific effects, but the adverse effect of Na^+^ and K^+^ salts on growth is related to the general effect of surplus salinity, possibly, due to high electrolyte activity. It has been shown, at least, for glycophyte species, that the negative effect of salinity is related to osmotic stress [46], but several studies have shown that treatment with isoosmotic concentration of organic osmolytes (as sorbitol or polyethylene glycol) result in more negative effect on halophyte growth in comparison to Na^+^ or K^+^ salts [47]. In fact, presence of ions can alleviate negative consequence of osmotic stress on plants [48]. However, it is reasonable to suggest that particularly anionic component of salts plays an important role in determining the nature of salinity responses. For example, both carbonate and hydrogen carbonate of Na^+^ have been shown to be more harmful in comparison to chloride form, which has been associated mainly with the alkalinity of these salts [10,49]. However, the role of chloride itself in salinity tolerance of halophytes has been recently questioned, and it was concluded that in the case of halophytes, Cl^–^ represents an essential osmoticum [50]. On the other hand, in halophyte species *Sesuvium portulacastrum* NaCl toxicity has been fully attributed to negative effects of Cl^–^ [45].

### 4.4. Osmotic Adjustment

According to the generally accepted mechanism of salinity tolerance in halophytes, Na^+^ and Cl^–^ are compartmented in vacuoles while osmotic balance is maintained by accumulation of organic solutes in cytoplasm [51]. Role of organic osmolytes in environmental stress tolerance has been shown also for species of genus *Armeria*. In high mountain specialist species, including *Armeria caespitosa*, both osmotically active carbohydrates and proline simultaneously act as osmoregulators in drought conditions [52]. Early studies with *A. maritima* have shown that proline accumulates in plant tissues as a result of salinity treatment [53]. There is no doubt that tissue proline concentration increases with increasing salinity or under the effect of other adverse environmental conditions in many species, but its contribution to osmotic control could be relatively small. Instead, a role of proline as a regulator of plant defense responses has been considered recently [54]. In addition, later it has been shown that *A. maritima* contain betaines [55] and even respond to increasing salinity by accumulation of betaine [35]. However, the role of “compatible solutes” in osmotic adjustment has been seriously questioned [56]. According to this analysis, it appears that inorganic osmolytes (K^+^, Na^+^, Cl^–^) are responsible for most of osmotic adjustment both in halophytes as well as glycophytes. In *A. maritima*, degree of adjustment by inorganic ions vs. organic osmolytes varied between NaCl and KCl treatments and different accessions, as well as between different plant parts, (Figure 10). Non-ionic component of osmotic value was relatively high in old leaves (about 40% of the total value) and significantly increased under NaCl treatment, especially for AM2 and AM3 plants at moderate salinity, but in AM1 only at high salinity (Figure 10A). In contrast, it significantly decreased in old leaves of AM2 plants treated with increasing concentration of KCl (Figure 10D). It has been also shown that for succulent halophytes, as *Salicornia bigelovii*, contribution of K^+^ to osmotic regulation is low in comparison to that of Na^+^ [57].

In Na-accumulating halophyte species, Na^+^ is stored in vacuoles of mesophyll cells [51], and there is an experimental evidence that surplus K^+^ is also stored in vacuoles, both regulating osmotic potential and acting as the main cellular reserve of K^+^ [58]. Earlier it was shown that *A. maritima* plants from salt marsh redistributed K^+^ from shoots to roots with increasing salinity [59], but no such response was found in the present study in spite of significant decrease of K^+^ concentration in leaves (Figure 7).

### 4.5. In Vitro vs. In Planta Effects

It has been a matter of long scientific debate if plant salinity tolerance assessed in conditions of tissue culture fully reflects that in whole plants cultivated in soil-like substrate [23]. For six halophytic species, higher tolerance to NaCl was evident in in vivo conditions, in comparison to in vitro culture [32]. In vitro cultivated explants of two out of six species could not propagate and survive at 400 mM NaCl, but the other species showed drastic inhibition of growth and development. In contrast, all plants survived 600 mM NaCl treatment when cultivated in substrate. This phenomenon was confirmed also in the present study, as multiplication intensity of *A. maritima* accessions AM1 and AM2 was negatively affected by increasing NaCl concentration (Figure 1B), and also biomass of AM2 decreased (Figure 1A) in conditions of tissue culture, but total biomass of both accessions was not significantly negatively affected by increasing salinity when cultivated in substrate (Figure 3). One of the reasons for this difference could possibly be related to root system acting as a barrier for Na^+^ transfer to shoots. Stimulated development of Casparian strips in roots under salinity can act as a barrier for passive apoplastic flux of Na^+^, resulting in a large concentration gradient between roots and shoots [60,61], similar to the limitation of heavy metal uptake [62]. However, this clearly was not the case in the present study, as roots of plant grown in soil accumulated much lower concentration of Na^+^ (Figure S2) in comparison to that in leaves (Figure 6), and Na^+^ concentration in cultivated shoot explants (≤1 mol kg^−1^, Figure 2A) at the highest salinity was similar to that in new leaves of soil-grown plants (Figure 6B).

## 5. Conclusions

It can be concluded that a species-wide salinity tolerance exists within dry sandy soil accessions of *A. maritima*, associated with the ability to accumulate surplus ions both in salt glands and old leaves. Both Na^+^ and K^+^ in a form of chloride salts had similar effect on plant growth, but with significant differences in osmotic adjustment by inorganic ions vs. organic osmolytes. Further studies aiming at dissecting physiological and biochemical mechanisms related to salinity tolerance of *A. maritima* and differences in osmotic and antioxidative protection of internal environment in different accessions due to various salinity types are necessary. Most importantly, functional diversity needs to be related to possible genetic diversity of different ecologically and geographically isolated micropopulations of *A. maritima* at the Northern range of distribution, to get further insight into abiotic stress adaptation mechanisms of the species.

## Figures and Tables

**Table 1:** Accessions of *Armeria maritima* used in the present study, characterization of their habitats and experiments performed.

Code	Associated Water Reservoir	Habitat	Electrical Conductivity (mS m^−1^)	Location	Coordinates	Performed Experiments (Treatments)
AM1	River Vecdaugava	Dry shore meadow	97 ± 5 c	City of Riga, Ziemeļu District, Vecdaugava, Latvia	57°03′29″ N 24°05′47′′ E	In vitro (NaCl 44, 110, 174, 217 mol L^−1^).In planta (NaCl 22, 44, 87, 217 mol L^−1^; KCl 22, 44, 87, 217 mol L^−1^)
AM2	River Buļļupe	Dry shore meadow	127 ± 4 b	City of Riga, Kurzeme District, Island of Buļļu Sala, Vakarbuļļi, Latvia	56°59′54″ N 23°57′31″ E	In vitro (NaCl 44, 110, 174, 217 mol L^−1^).In planta (NaCl 22, 44, 87, 217 mol L^−1^; KCl 22, 44, 87, 217 mol L^−1^)
AM3	The Baltic Sea	Dry coastal meadow	223 ± 11 a	Nybrostrand, Ystad Municipality, Skåne County, Sweden	55°25′40″ N 13°57′27″ E	In planta (NaCl 22, 44, 87, 217 mol L^−1^; KCl 22, 44, 87, 217 mol L^−1^); NaCl + KCl 44, 87, 217 mol L^−1^)

Different letters for soil electrical conductivity indicate statistically significant differences (*p* < 0.05).

**Table 2:** Effect of salinity treatment on morphological parameters of *Armeria maritima* accessions AM1 and AM2 cultivated for 8 weeks in soil.

Salt	Concentration (mmol L^−1^)	Flower Stalks (*n*)		Total Length of Flower Stalks (m Plant^−1^)		Dry Mass of Leaves (g)	
		AM1	AM2	AM1	AM2	AM1	AM2
Control	0	8.0 ± 0.7 abc	8.4 ± 1.0 ab	1.77 ± 0.14 abc	2.00 ± 0.25 ab	6.26 ± 0.47 ab	3.77 ± 0.52 a
NaCl	22	13.2 ± 1.2 a	9.8 ± 1.3 a	2.52 ± 0.30 a	2.13 ± 0.25 ab	7.64 ± 0.56 a	3.70 ± 0.35 a
	44	10.4 ± 0.5 ab	7.6 ± 0.6 ab	1.91 ± 0.09 ab	1.71 ± 0.17 abc	6.88 ± 0.55 ab	3.58 ± 0.40 a
	87	9.8 ± 0.9 abc	7.8 ± 0.5 ab	1.76 ± 0.12 abc	1.61 ± 0.13 abc	6.65 ± 0.61 ab	3.36 ± 0.27 a
	217	6.2 ± 0.6 c	6.6 ± 0.6 ab	1.02 ± 0.06 c	1.18 ± 0.16 bc	5.59 ± 0.30 ab	3.75 ± 0.37 a
KCl	22	10.8 ± 0.6 ab	10.0 ± 0.7 a	2.07 ± 0.15 a	2.26 ± 0.15 a	7.61 ± 0.75 a	3.66 ± 0.23 a
	44	9.8 ± 1.2 abc	9.6 ± 0.7 ab	2.07 ± 0.17 a	2.16 ± 0.20 a	6.73 ± 0.62 ab	3.66 ± 1.02 a
	87	9.4 ± 1.3 abc	8.4 ± 1.3 ab	1.86 ± 0.26 ab	1.89 ± 0.32 abc	6.48 ± 0.20 ab	4.13 ± 0.69 a
	217	7.6 ± 0.7 bc	5.4 ± 1.0 b	1.16 ± 0.13 bc	1.00 ± 0.21 c	5.06 ± 0.28 b	3.61 ± 0.26 a

Data are means ± SE from 5 replicates. Different letters between treatments for a particular parameter for each accession separately indicate statistically significant differences (*p* < 0.05).

**Table 3:** Effect of salinity treatment on dry mass of generative parts and roots of *Armeria maritima* accessions AM1 and AM2 cultivated for 8 weeks in soil.

Salt	Concentration (mmol L^−1^)	Dry Mass of Flower Stalks (g)		Dry Mass of Flowers (g)		Dry Mass of Roots (g)	
		AM1	AM2	AM1	AM2	AM1	AM2
Control	0	0.70 ± 0.03 abc	0.97 ± 0.06 abc	0.83 ± 0.08 ab	1.01 ± 0.05 ab	1.79 ± 0.20 a	1.18 ± 0.14 ab
NaCl	22	1.01 ± 0.13 a	1.13 ± 0.11 a	1.30 ± 0.17 a	1.08 ± 0.12 ab	1.93 ± 0.10 a	1.01 ± 0.19 ab
	44	0.80 ± 0.05 ab	0.89 ± 0.13 abc	1.03 ± 0.06 ab	0.95 ± 0.16 ab	1.73 ± 0.15 a	1.18 ± 0.14 ab
	87	0.68 ± 0.05 abc	0.89 ± 0.09 abc	0.99 ± 0.08 ab	1.04 ± 0.11 ab	1.19 ± 0.09 bc	0.93 ± 0.11 ab
	217	0.43 ± 0.01 c	0.57 ± 0.09 bc	0.79 ± 0.06 b	0.69 ± 0.05 b	0.75 ± 0.07 c	0.60 ± 0.03 b
KCl	22	0.91 ± 0.06 ab	1.33 ± 0.10 a	1.11 ± 0.08 ab	1.46 ± 0.11 a	1.44 ± 0.18 ab	1.45 ± 0.22 a
	44	0.87 ± 0.08 ab	1.07 ± 0.04 ab	1.13 ± 0.08 ab	1.14 ± 0.04 ab	1.51 ± 0.11 ab	1.12 ± 0.20 ab
	87	0.82 ± 0.12 ab	0.96 ± 0.20 abc	1.13 ± 0.16 ab	0.98 ± 0.14 ab	1.16 ± 0.07 bc	0.88 ± 0.03 ab
	217	0.58 ± 0.07 bc	0.49 ± 0.10 c	1.06 ± 0.08 ab	0.63 ± 0.13 b	0.77 ± 0.06 c	0.56 ± 0.05 b

Data are means ± SE from 5 replicates. Different letters between treatments for a particular parameter for each accession separately indicate statistically significant differences (*p* < 0.05).

**Table 4:** Effect of salinity treatment on morphological parameters of *Armeria maritima* accession AM3 cultivated for 7 weeks in soil.

Salt	Concentration (mol L^−1^)	Flower Stalks (*n*)	Total Length of Flower Stalks (m)	Dry Mass of Leaves (g)	Dry Mass of Flower Stalks (g)	Dry Mass of Flowers (g)	Dry Mass of Roots (g)
Control	0	10.6 ± 0.7 a	4.34 ± 0.26 a	2.63 ± 0.16 a	3.50 ± 0.14 a	2.64 ± 0.11 a	2.28 ± 0.34 ab
NaCl	22	8.2 ± 0.9 ab	3.80 ± 0.33 ab	3.11 ± 0.60 a	2.80 ± 0.31 ab	2.39 ± 0.18 abc	2.93 ± 0.49 a
	44	7.2 ± 0.6 abc	2.49 ± 0.24 bc	2.36 ± 0.33 a	1.77 ± 0.17 bcd	1.74 ± 0.18 abcde	1.38 ± 0.34 bcd
	87	5.4 ± 0.5 bc	1.81 ± 0.27 cd	2.16 ± 0.38 a	1.07 ± 0.24 cde	1.29 ± 0.40 abcde	1.02 ± 0.22 bcd
	217	4.5 ± 1.2 bc	1.19 ± 0.29 d	1.91 ± 0.20 a	0.65 ± 0.19 de	0.83 ± 0.26 de	1.13 ± 0.21 bcd
KCl	22	10.6 ± 1.1 a	4.03 ± 0.44 ab	2.74 ± 0.11 a	2.83 ± 0.23 ab	2.59 ± 0.35 ab	1.87 ± 0.39 abc
	44	7.4 ± 1.8 ab	2.51 ± 0.68 bc	2.36 ± 0.20 a	1.69 ± 0.44 cd	1.97 ± 0.60 abcde	1.23 ± 0.20 bcd
	87	5.4 ± 0.9 bc	1.69 ± 0.40 cd	2.27 ± 0.61 a	1.05 ± 0.27 cde	1.23 ± 0.29 bcde	0.90 ± 0.20 cd
	217	2.8 ± 0.7 c	0.64 ± 0.22 d	2.44 ± 0.64 a	0.39 ± 0.12 e	0.60 ± 0.18 e	0.82 ± 0.17 cd
NaCl + KCl	44	6.6 ± 0.8 abc	2.44 ± 0.17 bc	2.52 ± 0.26 a	1.97 ± 0.12 bc	2.18 ± 0.23 abcd	1.05 ± 0.12 bcd
	87	4.4 ± 0.3 bc	1.44 ± 0.37 cd	2.25 ± 0.26 a	0.92 ± 0.03 cde	1.00 ± 0.16 cde	0.61 ± 0.11 cd
	217	4.4 ± 0.7 bc	1.26 ± 0.22 d	1.89 ± 0.15 a	0.74 ± 0.14 de	0.86 ± 0.14 de	0.48 ± 0.03 d

Data are means ± SE from 5 replicates. Different letters between treatments for a particular parameter indicate statistically significant differences (*p* < 0.05).

## Data Availability

All data reported here is available from the authors upon request.

## Supplementary Materials

The following supporting information can be downloaded at: https://www.mdpi.com/article/10.3390/plants11192570/s1, Figure S1: Typical *Armeria maritima* (AM2) individuals 6 weeks after full treatment with 217 mol L^−1^ NaCl (A) and 217 mol L^−1^ KCl (B) showing formation of salt crystals on surface of leaves and flower stems; Figure S2: Effect of added NaCl concentration in soil on accumulation of Na^+^ in roots (A) and flower stalks (B) of *Armeria maritima* plants from different accessions after 7–8 weeks of cultivation; Figure S3: Effect of added NaCl (A,B) and KCl (C,D) concentration in soil on accumulation of K^+^ in roots (A,C) and flower stalks (B,D) of *Armeria maritima* plants from different accessions after 7–8 weeks of cultivation; Figure S4: Effect of added NaCl (A,B) and KCl (C,D) concentration in soil on electrical conductivity in roots (A,C) and flower stalks (B,D) of *Armeria maritima* plants from different accessions after 7–8 weeks of cultivation; Figure S5: Effect of added NaCl (A–E) and KCl (F–J) concentration on summed concentration of Na^+^ + K^+^ in old leaves (A,F), new leaves (B,G), flowers (C,H), roots (D,I) and flower stalks (E,J) of *Armeria maritima* plants from different accessions after 7–8 weeks of cultivation; Figure S6: Effect of added NaCl (A–E) and KCl (F–J) concentration in K^+^: Na^+^ concentration ratio in old leaves (A,F), new leaves (B,G), flowers (C,H), roots (D,I) and flower stalks (E,J) of *Armeria maritima* plants from different accessions after 7–8 weeks of cultivation; Figure S7: Effect of added NaCl (A,B) and KCl (C,D) concentration in soil on osmotic value in roots (A,C) and flower stalks (B,D) of *Armeria maritima* plants from different accessions after 7–8 weeks of cultivation; Figure S8: Effect of added NaCl (A,B) and KCl (C,D) concentration in soil on non-ionic osmotic value in roots (A,C) and flower stalks (B,D) of *Armeria maritima* plants from different accessions after 7–8 weeks of cultivation.
