# Supplementary material for: Salinity Tolerance, Ion Accumulation Potential and Osmotic Adjustment In Vitro and In Planta of Different Armeria maritima Accessions from a Dry Coastal Meadow
Source: Plants (Basel). 2022 Sep 29;11(19):2570. doi: 10.3390/plants11192570 (PMC9571588; doi:10.3390/plants11192570)
Supplement: Supplementary file 1 [file plants-11-02570-s001.zip › plants-1935956-supplementary.pdf]

Supplementary Material

Salinity Tolerance, Ion Accumulation Potential and Osmotic Adjustment In Vitro and In Planta of Different *Armeria maritima* Accessions from a Dry Coastal Meadow

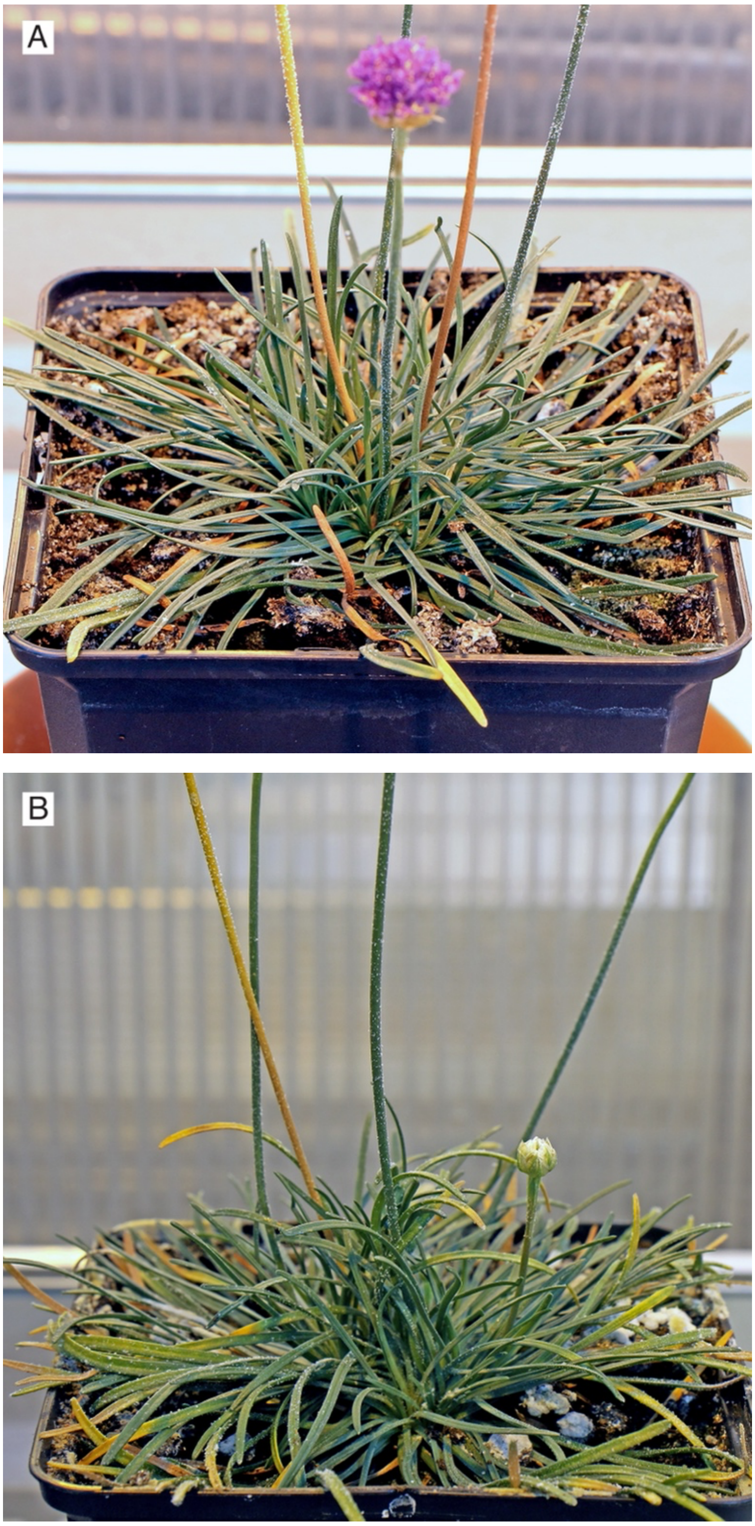

Figure S1. Typical *Armeria maritima* (AM2) individuals 6 weeks after full treatment with 217 mol L^{-1} NaCl (**A**) and 217 mol L^{-1} KCl (**B**) showing formation of salt crystals on surface of leaves and flower stems.

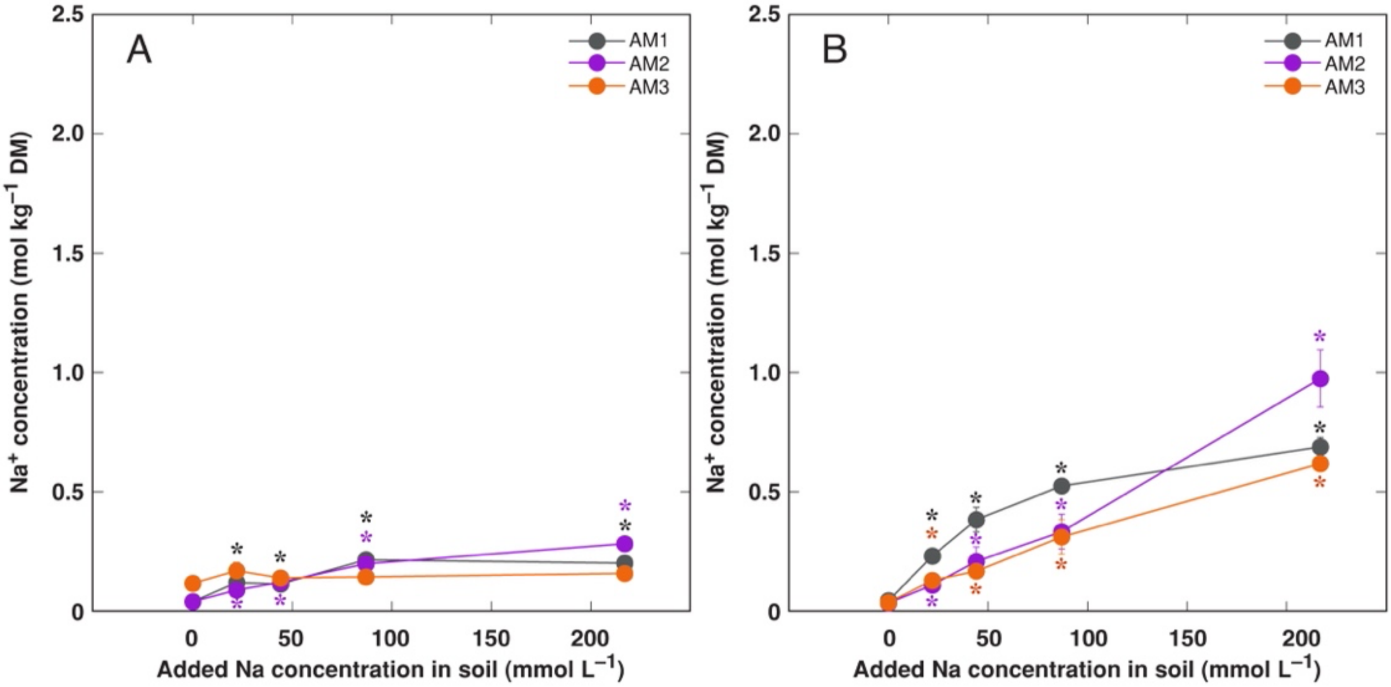

Figure S2. Effect of added NaCl concentration in soil on accumulation of Na^+ in roots (**A**) and flower stalks (**B**) of *Armeria maritima* plants from different accessions after 7–8 weeks of cultivation. Data are means ± SE from 3 replicates. Asterisks of respective color indicate statistically significant differences ($p < 0.05$) from control.

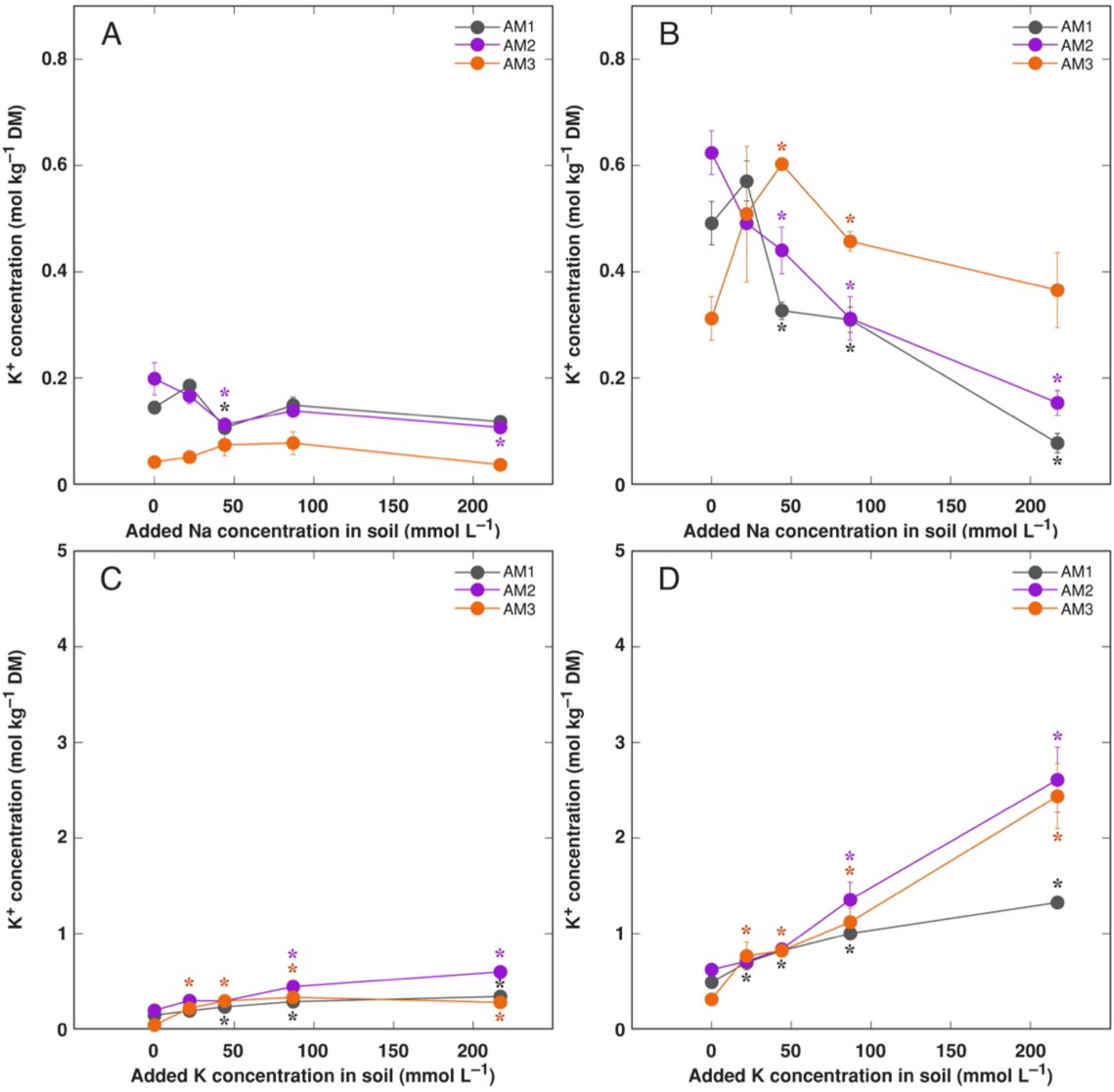

Figure S3. Effect of added NaCl (**A, B**) and KCl (**C, D**) concentration in soil on accumulation of K^+ in roots (**A, C**) and flower stalks (**B, D**) of *Armeria maritima* plants from different accessions after 7–8 weeks of cultivation. Data are means ± SE from 3 replicates. Asterisks of respective color indicate statistically significant differences ($p < 0.05$) from control.

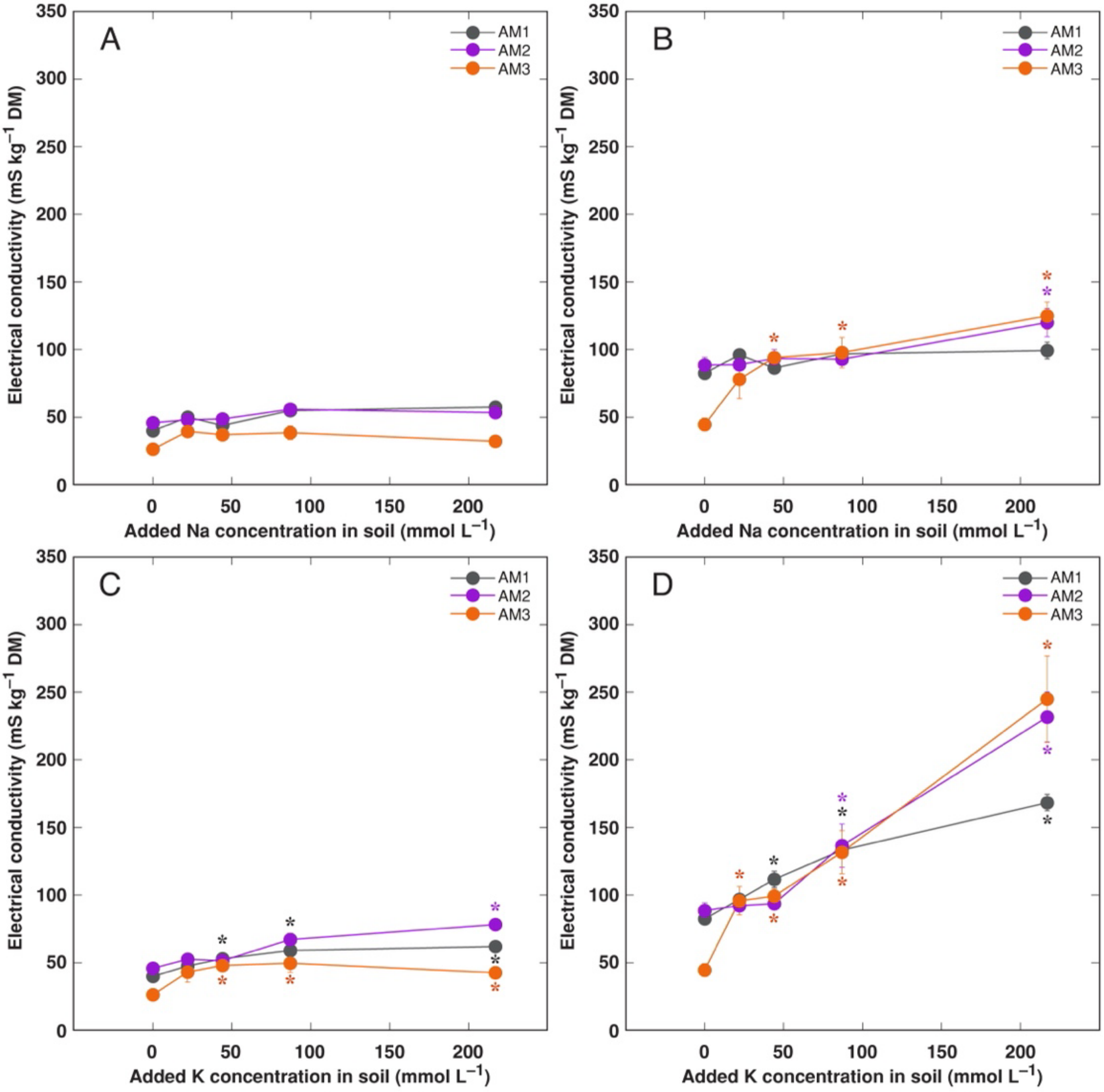

Figure S4. Effect of added NaCl (**A, B**) and KCl (**C, D**) concentration in soil on electrical conductivity in roots (**A, C**) and flower stalks (**B, D**) of *Armeria maritima* plants from different accessions after 7–8 weeks of cultivation. Data are means ± SE from 3 replicates. Asterisks of respective color indicate statistically significant differences ($p < 0.05$) from control.

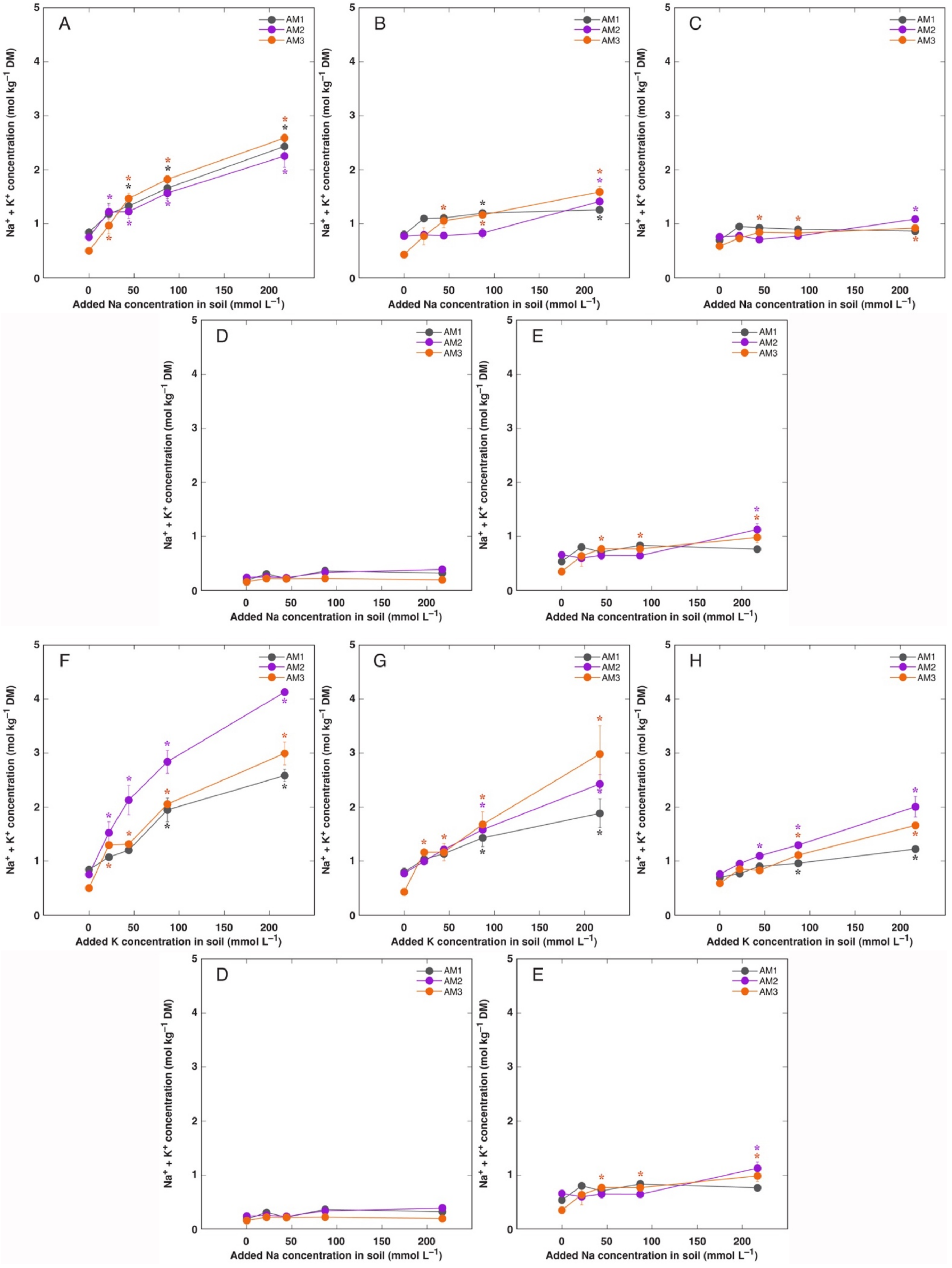

Figure S5. Effect of added NaCl (**A–E**) and KCl (**F–J**) concentration on summed concentration of Na^+ + K^+ in old leaves (**A, F**), new leaves (**B, G**), flowers (**C, H**), roots (**D, I**) and flower stalks (**E, J**) of *Armeria maritima* plants from different accessions after 7–8 weeks of cultivation. Data are means ± SE from 3 replicates. Asterisks of respective color indicate statistically significant differences ($p < 0.05$) from control.

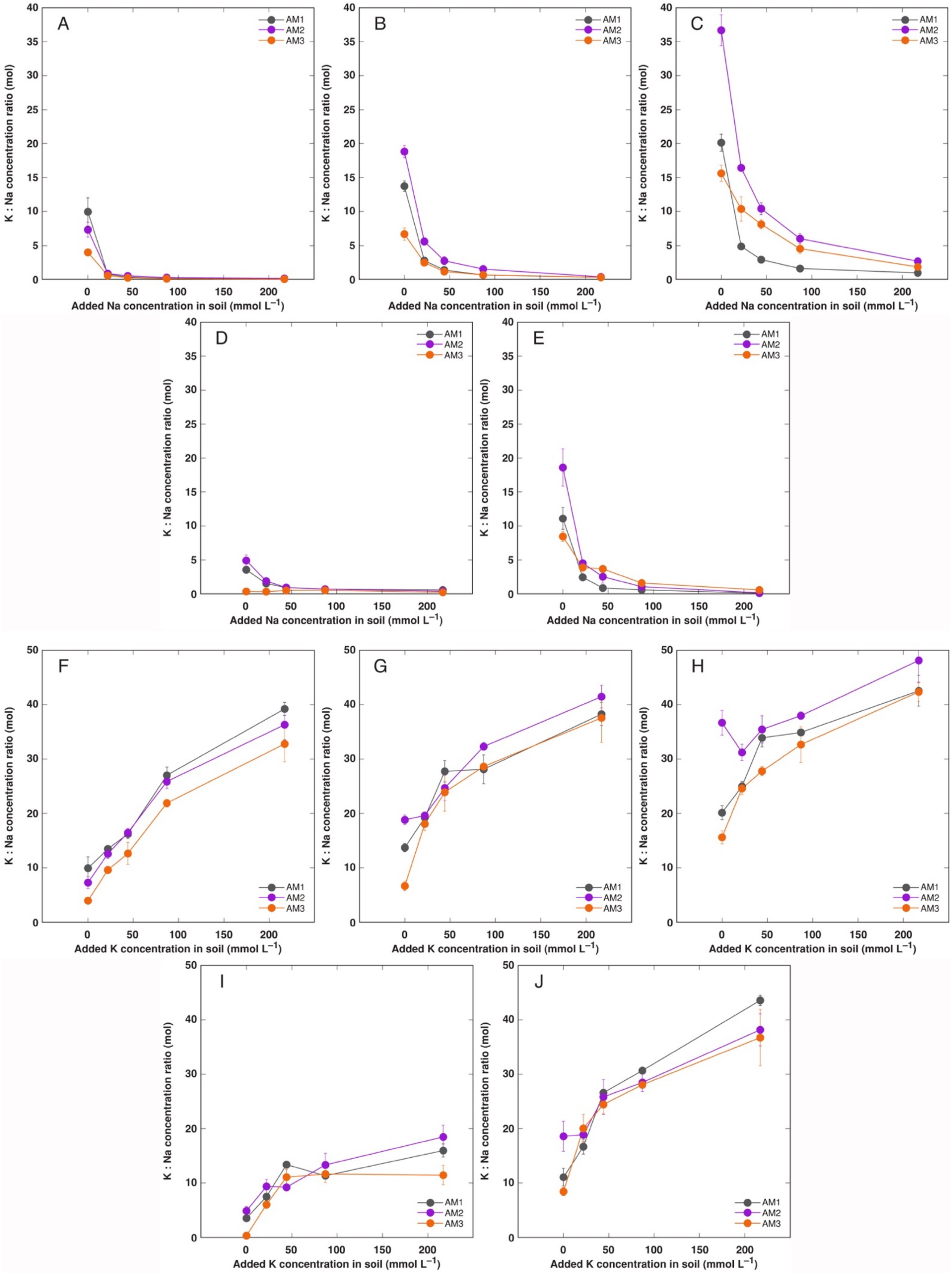

Figure S6. Effect of added NaCl (**A–E**) and KCl (**F–J**) concentration in K^+ : Na^+ concentration ratio in old leaves (**A, F**), new leaves (**B, G**), flowers (**C, H**), roots (**D, I**) and flower stalks (**E, J**) of *Armeria maritima* plants from different accessions after 7–8 weeks of cultivation. Data are means ± SE from 3 replicates. Asterisks of respective color indicate statistically significant differences ($p < 0.05$) from control.

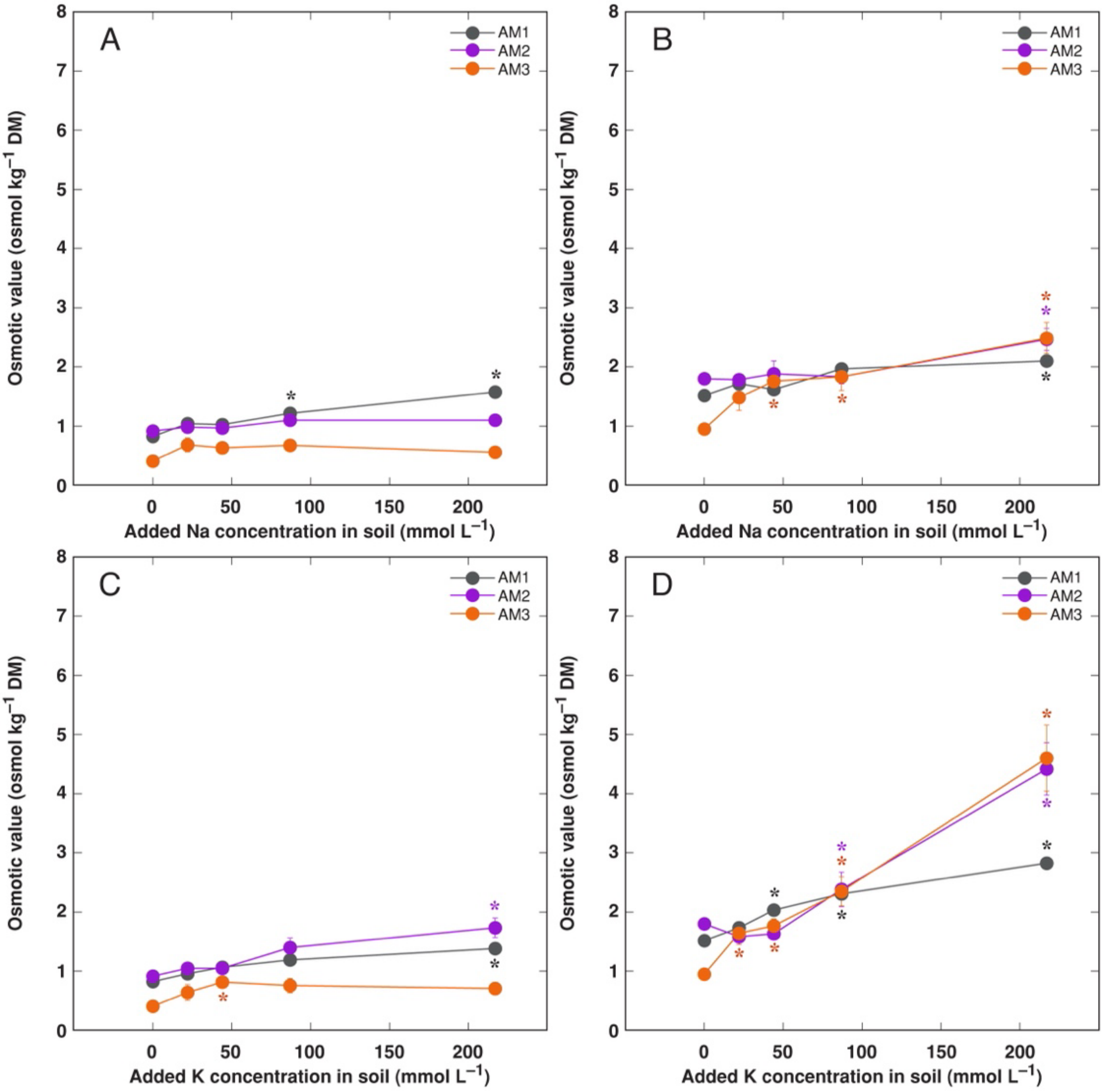

Figure S7. Effect of added NaCl (**A, B**) and KCl (**C, D**) concentration in soil on osmotic value in roots (**A, C**) and flower stalks (**B, D**) of *Armeria maritima* plants from different accessions after 7–8 weeks of cultivation. Data are means ± SE from 3 replicates. Asterisks of respective color indicate statistically significant differences ($p < 0.05$) from control.

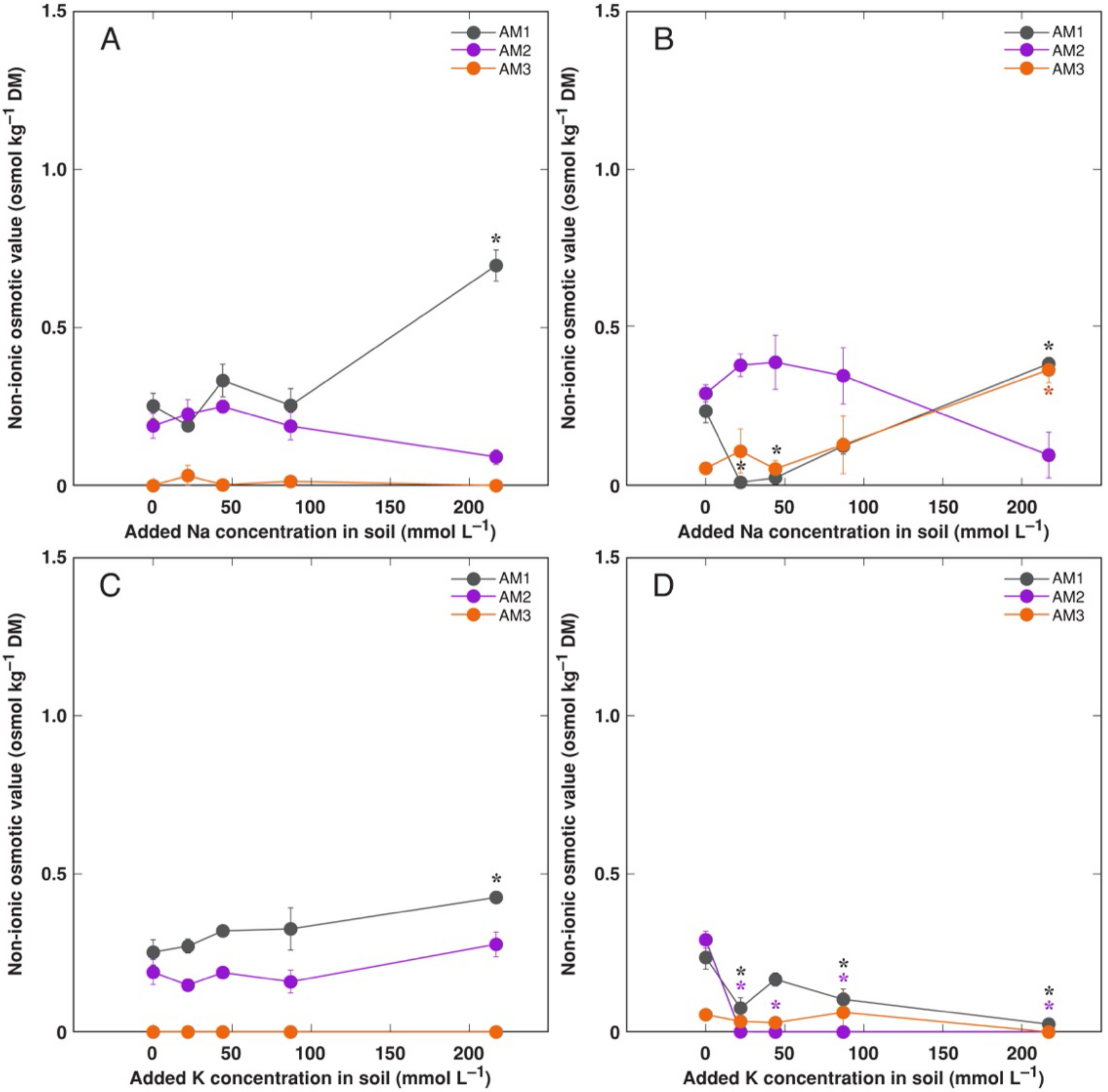

Figure S8. Effect of added NaCl (**A, B**) and KCl (**C, D**) concentration in soil on non-ionic osmotic value in roots (**A, C**) and flower stalks (**B, D**) of *Armeria maritima* plants from different accessions after 7–8 weeks of cultivation. Data are means ± SE from 3 replicates. Asterisks of respective color indicate statistically significant differences ($p < 0.05$) from control.
